# Biomechanical Differences in Bilateral Lower Limb Movement During the Back Kick Technique of Outstanding Taekwondo Athletes

**DOI:** 10.3390/life15121822

**Published:** 2025-11-28

**Authors:** Qinjian Xu, Hongwei Yan, Junli Yang, Wei Shan

**Affiliations:** 1 Sport Coaching College, Beijing Sport University, Beijing 100084, China; 2 Laboratory of Sport Training of General Administration of Sport of China, Beijing 100084, China; 3 China Institute of Sports and Health, Beijing Sport University, Beijing 100084, China

**Keywords:** taekwondo, biomechanics, injury risk, inter-limb asymmetry

## Abstract

Background: The back kick is a key scoring technique in taekwondo, often exhibiting bilateral asymmetry in lower limb function. Understanding these differences is crucial for optimizing training and minimizing injury risk. Methods: This study recruited twelve elite taekwondo athletes to perform back kicks using both their dominant and non-dominant legs under standardized conditions. Kinematic, kinetic, and surface electromyographic data were synchronously collected using a 3D motion capture system, force plate, and sEMG sensors. Paired *t*-tests and effect sizes assessed bilateral differences. Results: During the leg-lifting phase (P1), attacking leg peak hip power was significantly greater on the non-dominant side (*p* < 0.01); knee flexion angle was greater on the dominant side (*p* < 0.01), yet peak knee power was higher on the non-dominant side (*p* < 0.01). Support leg knee flexion angle was greater on the dominant side (*p* < 0.01), while knee flexion torque was higher on the non-dominant side (*p* < 0.05); ankle extension moment (*p* < 0.05) and plantar flexion power (*p* < 0.01) favored the dominant side. In the kicking phase (P2), dominant knee power was significantly higher (*p* < 0.01). The biceps femoris on the non-dominant side showed significantly higher iEMG and RMS values (*p* < 0.05), and dominant striking speed was faster (*p* < 0.05). Conclusions: These findings confirm marked functional asymmetry, suggesting training should emphasize non-dominant leg development to improve performance and reduce injury risk.

## 1. Introduction

Taekwondo is an Olympic competitive sport centered on leg techniques, integrating strength, speed, agility, and tactical judgment while offering both spectator appeal and practical combat utility [1,2]. In competitive matches, leg techniques serve as the primary scoring methods [3]. The back kick technique, valued for its stealth, high impact, and significant scoring potential [4] is widely employed in counterattack tactics during combat and serves as a key indicator of an athlete’s technical proficiency. Its execution relies on rapid hip rotation, stable trunk positioning, and linear footwork movement. The execution of a powerful and efficient back kick relies heavily on the coordination of several key lower-limb muscle groups, including the gluteus maximus, biceps femoris, rectus femoris, and gastrocnemius, which together contribute to hip extension, knee flexion and extension, and ankle plantarflexion. These muscle groups are critical in generating sufficient force, maintaining joint stability, and coordinating the rapid transition between the knee-lift and kicking phases. Prior electromyographic and biomechanical research indicates that asymmetries in the recruitment or performance of these muscles can influence both the technical effectiveness and injury risk associated with back kick execution [5,6,7,8]. Compared to techniques like roundhouse kicks or side kicks, back kicks deliver greater impact but demand superior spatial coordination and offer limited attack visibility. Despite ongoing revisions to Taekwondo competition rules that have increased back kick scoring values and usage frequency, effective scoring rates have not significantly improved [9].

Due to variations in training intensity and lower limb function, taekwondo athletes develop a dominant and non-dominant side of the lower body. The leg frequently used to attack opponents is defined as the dominant leg [10]. Through prolonged training and competition, taekwondo athletes typically perform a high volume of repetitive movements using their dominant side. This “unilateral dominance” in specialized training leads to pronounced lateral usage habits in the lower limbs, resulting in bilateral differences in muscle strength, joint flexibility, and joint angles. Lower limb asymmetry refers to performance disparities between both sides during movement [11], which can be classified based on dominant/non-dominant [12] or strong/weak side [13] characteristics. Taekwondo athletes adopt either the front stance or back stance, which influences leg function during kicking. Specific stances may foster habitual preference for one leg as the primary attacking side, establishing asymmetrical movement patterns [14,15]. However, this preference does not necessarily align with hand dominance. In competitive taekwondo, attack speed and accuracy are closely linked to movement characteristics of the hip, knee, and ankle joints [16,17,18]. For outstanding taekwondo athletes, moderate limb asymmetry aids specialized technical performance. Yet excessive asymmetry not only compromises the stability and coordination of technical movements but also increases energy expenditure and alters neuromuscular recruitment patterns on the non-dominant side [13]. Over time, these biomechanical imbalances may lead to reduced efficiency, technical errors, and a higher risk of overuse injuries, potentially impacting competition outcomes [19,20].

Although the relationship between lower limb asymmetry and athletic performance has received extensive attention, existing research has primarily focused on running, jumping [21], and sports requiring bilateral coordination, such as track and field [22] and cycling [11], with limited attention given to highly specialized technical sports like taekwondo. Current taekwondo research predominantly centers on biomechanical analysis of back kicks [23], yet critical differences in kinematics, dynamics, and electromyographic parameters between the supporting and striking legs remain under-explored. Specifically, the precise impact of these differences on technical execution effectiveness remains unclear. Notably, recent studies indicate that not all taekwondo techniques exhibit pronounced lateralization. Góra et al. (2024) observed no significant differences in muscle strength symmetry between the left and right lower limbs during certain linear techniques in traditional taekwondo athletes [24]. This suggests that movement type may partially determine the presence and intensity of lateralization. Liu et al. (2023) employed an integrated experimental platform combining the Vicon motion capture system and Kistler 3D force plates to simultaneously collect three-dimensional kinematic and kinetic data. This enabled the analysis of lateralization phenomena in the front kick movement, ultimately confirming that the dominant side exhibits significant advantages in both force output and muscle activation efficiency [10,25]. Isaac Estevan (2015) quantified activation patterns of core and lower-limb key muscle groups during different Taekwondo kicking techniques using surface electromyography (sEMG) [5]. This provided comparable, reliable evidence of muscle recruitment patterns for the neuromuscular control mechanisms underpinning movement technique. These studies validate the practical value of this integrated system in revealing the multidimensional ‘strength-technique-neurology’ mechanisms underlying athletic performance. Therefore, this study employs an integrated 3D motion capture + force plate + surface EMG system to achieve synchronous, quantitative, and temporal assessment of key biomechanical characteristics in taekwondo back kicks. These include lower limb multi-joint coordination, ground reaction force variations, and muscle activation timing. This facilitates comprehensive identification of the functional roles and interrelationships between the supporting and attacking legs during technical execution. Thus, this study aims to investigate the differential characteristics in kinematic, dynamic, and surface electromyographic (sEMG) indicators between the bilateral lower limbs during the execution of the back kick by elite taekwondo athletes, and to analyze their potential underlying mechanisms in technical performance. We hypothesize that: (1) the dominant leg will exhibit greater peak joint torque, angle, power, and kicking speed compared to the non-dominant leg; (2) significant differences in electromyographic activity will be observed between the bilateral lower limbs, with higher activation on the non-dominant side reflecting compensatory neuromuscular strength.

## 2. Materials and Methods

### 2.1. Research Subjects

Using G*Power 3.1.9.7 software for sample size estimation, a priori analysis was performed for a two-tailed paired-sample *t*-test [7,26,27], with an effect size d = 0.7, significance level α = 0.05, and statistical power (1-β) of 80%. The results indicate that a minimum of 11 participants is required [28,29]. Twelve national-level and above taekwondo athletes (six males and six females), all black belt holders, were recruited as research subjects through questionnaires and posters. Participants had an average age of 19.19 ± 1.91 years, height of 1.77 ± 0.10 m, weight of 63.38 ± 9.05 kg, and training duration of 8.38 ± 2.75 years. The group comprised 1 national champion athlete and 11 first-class athletes, with 3 left-leg dominant and 9 right-leg dominant athletes. This study examined differences in lower-limb biomechanical indicators between the left and right sides during the back kick technique of outstanding athletes. Although some studies suggest that gender may influence technical performance in specific sports, the impact of gender differences on taekwondo techniques remains controversial. Góra et al. conducted a kinematic analysis of side kicks and spinning kicks in taekwondo athletes, finding no statistically significant differences in effective mass metrics between male and female athletes [24]. This suggests that movement technique may be a more critical determinant than gender. Accordingly, the present study employed standardized technical parameter analysis without grouping by gender.

Inclusion Criteria: Athletes aged 18–25 years; in good health with no history of cardiac or neuromuscular disorders; participating in an average of 5 taekwondo training sessions per week, each lasting at least 3 h; participants engaged in an average of 5 taekwondo training sessions per week, each lasting at least 3 h. Training routines typically consisted of dynamic warm-ups, fundamental kicking drills, tactical sparring, resistance training (1–2 sessions/week), and flexibility or recovery sessions. Athletes followed individualized training plans supervised by national-level coaches, ensuring consistent technical refinement and physical conditioning; no high-intensity training within 24 h prior to testing; no lower limb joint injuries within the preceding 6 months; and normal physical condition and athletic performance [9].

Exclusion Criteria: Lower limb joint injuries within the past 6 months; High-intensity exercise within 24 h prior to testing; Respiratory, cardiovascular, or hematological disorders.

All subjects signed written informed consent forms. All procedures were conducted in accordance with the Declaration of Helsinki and approved by the local ethics committee (Reviewing Body: Beijing Sport University Experimental Ethics Committee for Sports Science; Approval No. 2025065H).

### 2.2. Research Methods

#### 2.2.1. Test Procedure

Kinematic, dynamic, and surface electromyography data were collected using the Vicon 3D motion capture system (Vicon, Lachine, QC, Canada, Sampling frequency: 200 Hz), Kistler 3D force platform (Kistler, Winterthur, Switzerland, Sampling frequency: 1000 Hz), and Zhiyunwei electromyography acquisition device (Zhiyunwei, Shanghai, China, Sampling frequency: 1000 Hz). The subjects’ height and weight were measured using the HC Height and Weight Measuring Device (HC, Hongkong, China, Accuracy: ±0.1 cm, ±0.1 kg).

The experiment was conducted in a biomechanics laboratory with an average temperature of 26 °C. Experimenters synchronized the electromyography (EMG) equipment with the Vicon motion capture system using a synchronization cable. Prior to the experiment, all subjects were instructed to warm up on a treadmill at 6.5 km/h [30] followed by stretching exercises and test movements. Before recording the back kick technique, subjects were required to expose their upper bodies while wearing black compression leggings on their lower bodies. Staff disinfected and removed hair from electrode placement sites using alcohol swabs. Skin abrasion paper was applied to remove the stratum corneum, followed by another alcohol swab disinfection to reduce impedance [31] and enhance electromyographic signal quality. After allowing the skin to air-dry, electrodes were applied. Bipolar electrode pads were affixed parallel to muscle fiber orientation at the mid-belly of the target muscle, spaced 2 cm apart. Electrodes were secured and calibrated to prevent dislodgement during testing. Subject information was entered into the computer system, and testing proceeded according to the experimental protocol [32].

Prior to the experiment, subjects underwent Vicon static calibration within the testing area. Electronic protective gear height and distance from subjects were adjusted according to individual stature. Athletes’ dominant legs were defined as their preferred leg for movement; three subjects in this study utilized their left leg as dominant, while the remainder used their right leg. Building upon the 39-point full-body model marker placement scheme provided by Vicon’s Plug-In Gait software (Vicon Motion Systems, Oxford Metrics, Yarnton, UK), additional tracking points were added to the existing bony landmarks at the hip, thigh, calf, and foot, tailored to the characteristics of taekwondo [10]. Furthermore, based on the technical characteristics of the back kick [8], during the execution of this technique, the kicking side primarily relies on the hip extensors (gluteus maximus, biceps femoris), the knee extensors (quadriceps femoris), and the ankle plantar flexors (gastrocnemius) to achieve rapid extension and impact output [5]. This study primarily focuses on surface electromyography (sEMG) parameter measurements of four muscles in the attacking leg of the back kick technique that are highly correlated with this movement. The electrode patches are attached to the muscles and the attachment positions are as follows: Gluteus maximus (Between the greater trochanter of the left/right femur and the sacral vertebrae), Rectus femoris muscle (The midpoint of the line connecting the anterior superior iliac spine and the upper end of the patella), Biceps femoris (Medial to the midline of the left/right posterior thigh, from the gluteal cleft to the midpoint of the knee joint), Gastrocnemius muscle (The middle third of the distance between the head of the left/right fibula and the heel) [33].

Meanwhile, the supporting leg side must maintain stability and transmit ground reaction forces, while core trunk muscles (such as the internal and external obliques, multifidus) are crucial for balance and rotational control during the movement [30,34]. Previous studies indicate that the supporting leg bears greater loads (e.g., vertical ground reaction force and muscle activation) during high-intensity movements, exhibiting lateralization in both kinematic and electromyographic patterns. This provides a theoretical foundation for investigating lower-limb functional asymmetry.

Participants first underwent simulated testing with their dominant and non-dominant sides to adapt to the positioning and dimensions of the three-dimensional force platform. During the formal testing phase, subjects initiated strikes against the electronic protective gear using their dominant and non-dominant sides, respectively. Two national-level taekwondo referees evaluated the subjects’ movements during each strike, with three valid data points collected per athlete.

#### 2.2.2. Data Processing

##### Action Phase Classification

**Figure 1:**
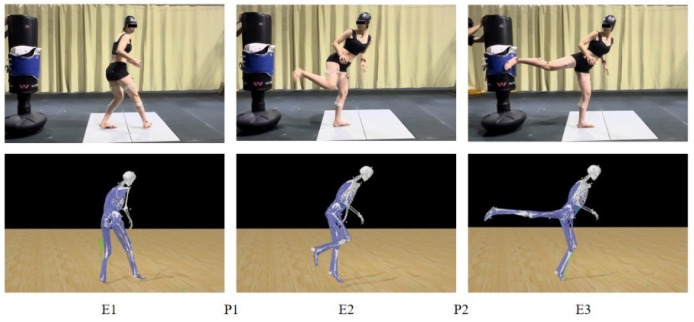
Timing and Phases of the Back Kick Technique.

According to research by Kim [8], Mijia Jin [35], and others, the back kick technique is divided into the knee lift phase (P1) and the kicking phase (P2). These phases are further subdivided into three distinct moments: the preparation moment (both feet on the ground, just before the attacking leg leaves the ground, denoted as E1), the knee-bending moment (when the knee is raised and the body rotates, with the smallest angle between the thigh and lower leg, denoted as E2), and the kicking moment (when the knee strikes the protective gear and the knee joint reaches its maximum extension angle, denoted as E3) (see Figure 1).

##### Data Analysis

The captured kinematic data were processed using the proprietary data processing software of the VICON 3D motion capture system (Oxford Metrics Ltd., Oxford, UK). First, a full-body 3D human skeleton model was constructed based on dynamically acquired marker points. Data integrity was restored through point connection and interpolation operations. Subsequently, the processed data was exported as a C3D format file and saved. The exported C3D file was imported into Visual3D software (C-Motion Inc., Ontario, Canada) for subsequent analysis. Kinematic parameters underwent low-pass filtering at a frequency of 10 Hz, while kinetic parameters were processed using a 25 Hz filter frequency.

Surface EMG data were processed using the EmgServer 3.0 software bundled with the surface EMG acquisition device. This involved rectifying the EMG signals and applying a fourth-order zero-phase Butterworth bandpass filter with a cutoff frequency range of 10–480 Hz. Subsequently, the filtered EMG signals underwent full-wave rectification and smoothing using a 50-millisecond moving RMS window.

Joint torques were calculated using Visual3D inverse dynamics; ground reaction force data underwent filtering, followed by division by the subject’s body mass and then by the gravitational acceleration constant g to achieve standardization; surface electromyography parameters were exported using the EmgServer 3.0 software bundled with the surface EMG acquisition device; joint power was obtained by multiplying joint torque and joint angular velocity. Therefore, the selected indicators in this study are:

Kinematics Metrics: Maximum flexion angle of the hip and knee joints during movement (unit: °); peak linear velocity of the foot along the vertical attack direction (unit: m/s).

Kinematic indicators: Peak ground reaction force in the vertical direction after normalization (unit: BW); maximum joint torques at hip, knee, and ankle (unit: Nm/kg) and maximum power output (unit: W/kg).

Surface electromyography indicators: Integral electromyogram (IEMG) (unit: μVs), root mean square amplitude (RMS) (unit: μV).

To ensure directional comparability of biomechanical indicators between the dominant and non-dominant lower limbs, the positive and negative signs for each study indicator were standardized to guarantee consistent directional definitions. Following the definitions for coordinate axes and joint angles in Visual3D software and human anatomy, hip flexion, hip abduction, knee extension, plantar flexion, and ankle external rotation were designated as positive directions; conversely, hip extension, knee flexion, and dorsiflexion were defined as negative directions. Foot movement velocity aligned with the direction of attack was considered positive; otherwise, it was negative [20,36,37].

##### Statistical Analysis

Statistical analyses were conducted using SPSS 27.0 software. Data were presented as mean ± standard deviation. Normality was assessed using the Shapiro–Wilk test. For normally distributed variables, paired *t*-tests were used to compare the dominant and non-dominant sides, and Cohen’s d effect sizes were calculated to quantify the magnitude of the differences. For non-normally distributed variables, the Wilcoxon signed-rank test was applied as a non-parametric alternative for paired data. A two-tailed *p*-value of <0.05 was considered statistically significant.

## 3. Results

### 3.1. Biomechanical Characteristics of the Hip Joint

**Figure 2:**
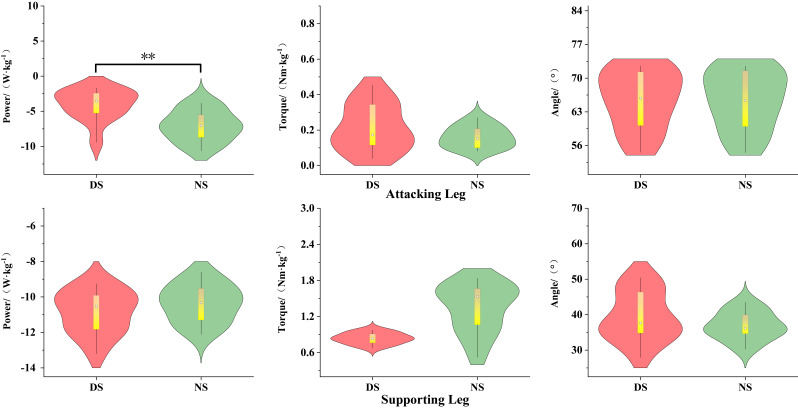
Biomechanical Differences in the Knee-Lifting Phase of Hip Joint Movement. Note: ** indicates *p* < 0.01.

**Figure 3:**
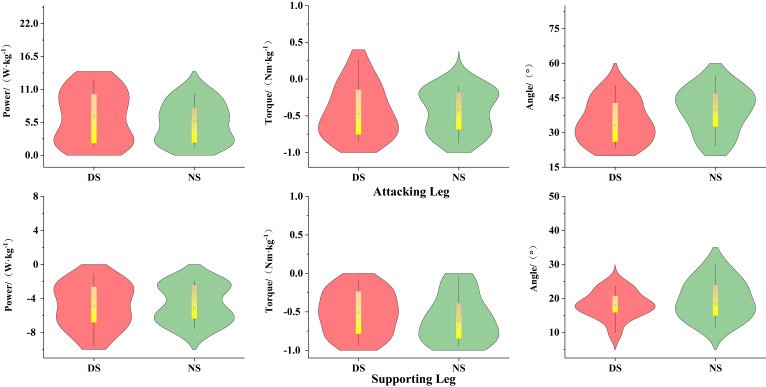
Biomechanical Differences in the Kicking Phase of the Hip Joint.

As shown in Figure 2, during the knee lift phase (P1), the peak power of the non-dominant hip joint was significantly greater than that of the dominant hip joint (*p* < 0.01, d = 1.34). As shown in Figure 3, there was no significant difference between the dominant and non-dominant sides (*p* > 0.05).

### 3.2. Biomechanical Characteristics of the Knee Joint

**Figure 4:**
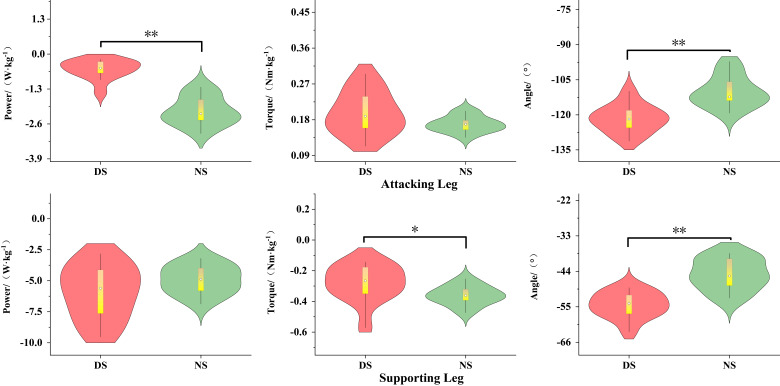
Biomechanical Differences in Knee-Lifting Phase Movement. Note: * indicates *p* < 0.05, ** indicates *p* < 0.01.

**Figure 5:**
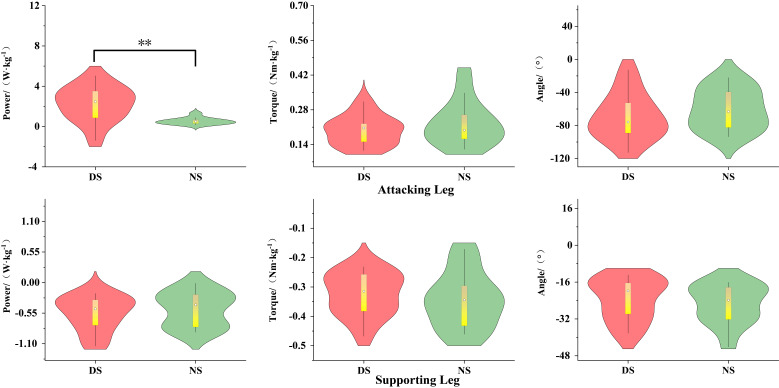
Biomechanical Differences in the Kicking Phase of the Knee Joint. Note: ** indicates *p* < 0.01.

As shown in Figure 4, during the knee lift phase (P1), the knee flexion angle of the dominant side attacking leg was significantly greater than that of the non-dominant side (*p* < 0.01, d = 2.17), while the peak knee power of the non-dominant side was significantly greater than that of the dominant side (*p* < 0.01, d = 2.51). The knee flexion angle of the support leg was significantly greater on the dominant side than on the non-dominant side (*p* < 0.001, d = 1.56), while the knee flexion torque was significantly greater on the non-dominant side than on the dominant side (*p* < 0.05, d = 0.62). As shown in Figure 5, during the kicking phase (P2), the peak power of the dominant leg was significantly higher than that of the non-dominant leg (*p* < 0.01, d = 0.96).

### 3.3. Biomechanical Characteristics of the Support Leg Ankle Joint

**Figure 6:**
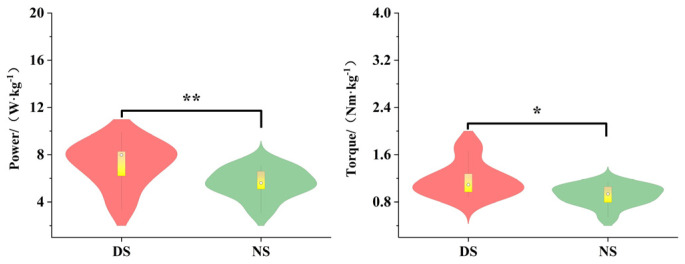
Biomechanical Differences in Ankle Joint Movement of the Support Leg During the Knee-Lifting Phase. Note: * indicates *p* < 0.05, ** indicates *p* < 0.01.

As shown in Figure 6, during the knee lift phase (P1), the ankle joint extension moment (*p* < 0.05, d = 0.81) and plantar flexion power (*p* < 0.01, d = 1.27) of the support leg were significantly greater on the dominant side than on the non-dominant side.

### 3.4. Surface Electromyography Characteristics

#### 3.4.1. Integral Electromyography (IEMG) Feature Analysis

**Figure 7:**
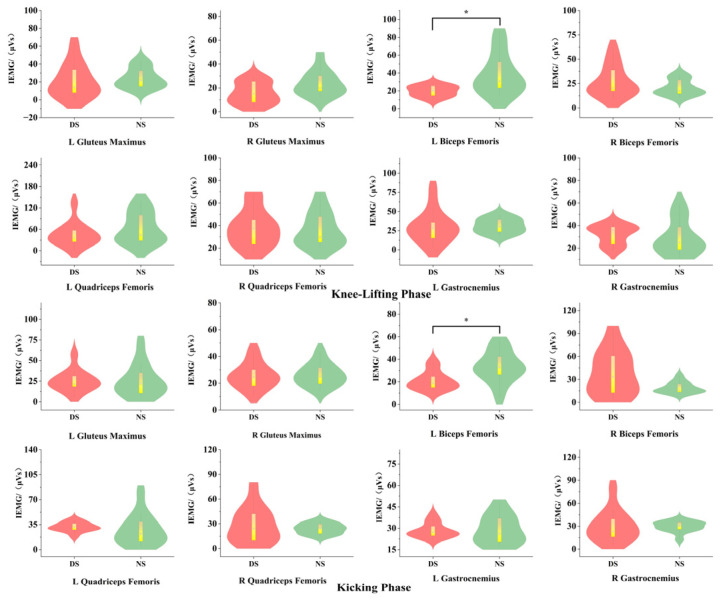
IEMG Difference Features. Note: * indicates *p* < 0.05.

As shown in Figure 7, during the knee-lifting phase (P1), significant differences existed between the dominant and non-dominant sides of the biceps femoris in integral electromyography (iEMG) values (*p* < 0.05, d = 0.69). During the kicking phase (P2), significant differences in integrated electromyography (iEMG) values were observed between the dominant and non-dominant sides of the left biceps femoris (*p* < 0.05, d = 0.69).

#### 3.4.2. Root Mean Square (RMS) Amplitude Analysis

**Figure 8:**
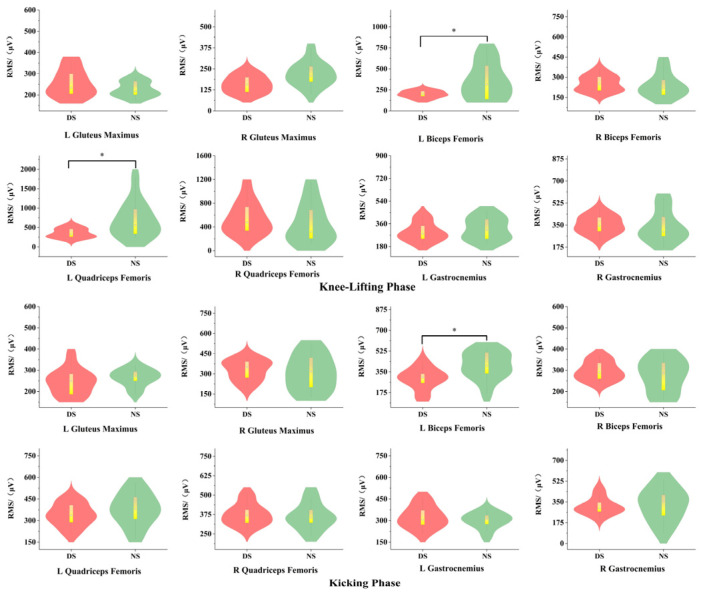
RMS Difference Features. Note: * indicates *p* < 0.05.

As shown in Figure 8, during the knee-lifting phase (P1), significant differences in RMS amplitude values were observed between the dominant and non-dominant sides for the left biceps femoris (*p* < 0.05, d = 0.73) and left quadriceps femoris (*p* < 0.05, d = 0.68). During the kicking phase (P2), the RMS amplitude of the left biceps femoris (*p* < 0.05, d = 0.82) exhibited significant differences between the dominant and non-dominant sides.

### 3.5. Striking Velocity and Vertical Ground Reaction Force Characteristics

As shown in Table 1, during the kicking phase (p2)—specifically when the attacking leg struck the protective gear (E3)—significant differences existed between the striking velocities of the dominant side (0.67 ± 0.12 m·s^−1^) and the non-dominant side (0.53 ± 0.08 m·s^−1^) (*p* < 0.01, d = 0.14).

## 4. Discussion

### 4.1. Kinematic and Kinetic Characteristics of the Lower Limb During the Knee-Lifting Phase

The knee-lifting phase (P1) serves as the foundational preparatory stage for the back kick technique. The attacking leg must rapidly flex at the hip and knee while completing hip abduction to establish the striking readiness posture. Joint torque refers to the rotational effect generated by forces acting on a joint, representing a key physical quantity describing joint motion and loading [38]. Joint power is defined as the amount of work performed by a joint per unit time, serving as a core metric for measuring energy output during joint movement [39]. This study found that the hip flexion moment and power output of the non-dominant attacking leg were significantly higher than those of the dominant side. This indicates that the dominant side exhibits more efficient performance in maintaining body stability, transmitting ground reaction forces upward, and assisting the attacking leg in knee lift and force generation. However, it also consumes more energy during movement execution, potentially due to insufficient neuromuscular control precision [40]. Although this compensatory force generation pattern can sustain movement completion in the short term, it leads to excessive fatigue in the hip joint and associated muscle groups over time, thereby reducing the attack speed of the back kick technique. Conversely, the dominant side shortens knee-lifting time through more efficient joint coordination. Fox et al. also noted that excessive lower-limb asymmetry significantly diminishes specialized technical performance and may even increase the risk of sports injuries [11]. Thus, lower-limb asymmetry negatively impacts taekwondo back kick performance. The knee-lifting phase during the back kick is closely correlated with knee flexion angle, a key indicator for assessing knee-lifting proficiency [41]. During the knee-lifting phase (P1), the knee flexion angle of the dominant attacking leg was significantly greater than that of the non-dominant side. Greater knee flexion facilitates rapid knee extension during kicking; faster knee extension speed correlates with relatively higher striking velocity [42], potentially reflecting more efficient utilization of the stretch-shortening cycle (SSC) mechanism on the dominant side. Rapid knee flexion accumulates elastic energy, creating conditions for greater kicking speed during subsequent rapid extension.

Electromyography (EMG) data revealed that the root mean square amplitude and integral value of the biceps femoris muscle during the knee lift phase were higher on the non-dominant side than on the dominant side. This indicates that the non-dominant side requires additional recruitment of motor units to complete the movement, consistent with the findings on hip joint torque and power output. This finding aligns with Schorderet et al.’s assertion that “the supporting leg serves not only as a fulcrum but also as a critical component for kinetic energy transfer and torque generation” [12]. Practical instruction and training should emphasize supporting leg conditioning and stability control to prevent it from becoming a technical bottleneck. Striking velocity, force, and leg technique symmetry all influence joint power output [23]. Regarding the supporting leg, this study found that the dominant side demonstrated significantly greater flexion angle and ankle plantar flexion force compared to the non-dominant side. This indicates higher ankle power output during the knee-lifting phase when the dominant leg serves as the support. Related research further suggests that in taekwondo kicks, the supporting leg not only regulates balance but also transfers energy to the kicking leg via ground reaction forces. This disparity further explains why athletes tend to execute back kicks with their dominant side in actual combat [9]. Therefore, optimizing technique during the knee-lifting phase requires not only focusing on kicking trajectory and angle control but also developing bilateral symmetry training programs that address multiple dimensions, including power chain integrity, neuromuscular control efficiency, and muscle strength balance.

### 4.2. Kinematic and Kinetic Characteristics of Lower Limb Movement During the Kicking Phase

The kicking phase (P2) is the critical stage for concentrated energy release in back kick techniques, determining both movement speed and scoring effectiveness. Its core principle involves multi-joint coordinated power generation through the hip-knee-ankle chain, achieving “proximal dominance and distal acceleration” to maximize terminal kick velocity. By adjusting attack distance, athletes can better adapt to opponents’ techniques and tactics during competition, thereby selecting appropriate scoring strategies. Changes in trunk and hip joint angles can regulate attack distance [43], while hip flexion and extension movements also influence attack distance [15]. This study found superior performance on the dominant side compared to the non-dominant side in metrics such as hip flexion force and attack speed. This phenomenon may stem from the non-dominant side compensating for reduced efficiency in the attacking leg by enhancing force output in the supporting leg when coordination is impaired. Huang et al. (2025) similarly observed in their study of taekwondo side kicks that the non-dominant side often increases energy output in the supporting leg to maintain overall movement continuity and stability [44]. Although the non-dominant side can complete movements through compensatory mechanisms, prolonged reliance on this pattern in competitive settings may increase kicking energy expenditure, preventing the body from sustaining efficient and stable performance [10]. Moderate asymmetry may result from individualized training, but when differences exceed specific thresholds, it becomes a key factor limiting the efficiency of specialized techniques [45].

Extension force refers to a muscle’s energy output capacity during the extension phase. During the kicking phase (P2), the dominant side’s attacking leg demonstrated higher knee extension force, indicating greater explosive power in the dominant side’s knee extensor muscles [46]. This enables the joint to generate greater power in a shorter timeframe, thereby enhancing the horizontal ground reaction force and propelling the back kick to complete the striking action at higher velocity [47]. This is crucial for improving the success rate of scoring actions. Previous studies indicate that knee extension power is significantly correlated with kicking speed, serving as a key determinant of striking force and hitting efficiency [48]. Furthermore, rapid knee extension enhances the horizontal ground reaction force in the lower limbs, thereby increasing terminal impact velocity. This aligns with the higher kicking speed demonstrated by the dominant side in this study [49], thereby providing athletes with a technical advantage. Liu et al. (2023) further confirmed in their double flying kick study that differences in joint power and terminal velocity were highly correlated with scoring performance on protective gear, while excessive asymmetry reduced scoring efficiency and increased joint load [10]. Therefore, competitive training should prioritize specialized strength and neuromuscular coordination training for the non-dominant side. Through bilateral load balancing and symmetrical technical movement interventions, limitations imposed by unilateral dominance on performance can be reduced, enhancing the sustainability and fatigue resistance of competitive techniques. For the kicking phase, focus should be placed on enhancing the explosive power and SSC utilization capacity of the non-dominant leg. Optimize its movement efficiency through elasticity training, rapid strength training, and neuromuscular control training [28]. Simultaneously, strengthen the supporting leg’s power and stability training to improve energy transfer and overall coordination. This enables more balanced bilateral performance and prevents the accumulation of sports injury risks caused by unilateralization.

### 4.3. Surface Electromyographic Characteristics of the Lower Limb

Electromyographic signals represent action potentials of muscle fiber motor units, reflecting the spatiotemporal superposition of muscle motor unit action potentials. They serve as a crucial indicator for evaluating neuromuscular recruitment efficiency and coordination patterns [50]. Integrated electromyography (IEMG) reflects the total discharge of a muscle over a specific time period, reflecting both the number of motor units participating in muscle work and their discharge intensity [33]. Surface EMG results indicate that during the knee lift phase (P1) and kick phase (P2), the integrated EMG values of the left biceps femoris were significantly higher on the non-dominant side than on the dominant side. This reflects more efficient neural control and movement economy on the dominant side, achieving the same action with lower EMG activation. Conversely, the non-dominant side requires more motor units and greater neural drive to execute the technical movement. This finding aligns closely with Bouhlel et al.’s (2015) assertion that “neuromuscular recruitment patterns directly determine technical movement efficiency” [51]. The results further confirm that the hip and knee joints of the non-dominant attacking leg require significantly higher joint power during the execution of the back kick technique compared to the dominant side.

The root mean square (RMS) value reflects muscle discharge efficiency and correlates with motor unit recruitment and synchrony of excitation rhythms. Optimizing muscle recruitment patterns can significantly enhance the speed and stability of taekwondo kicking techniques [52]. Conversely, the non-dominant side exhibits greater electromyographic fluctuations and activation time differences due to insufficient neuromuscular recruitment efficiency, indicating inadequate neuromuscular control stability. Notably, prolonged unilateral training exacerbates differences in bilateral lower limb muscle activation patterns, leading to functional asymmetry. Furthermore, this study found that during the knee-lifting phase (P1), the RMS values of both the left biceps femoris and left quadriceps femoris muscles on the non-dominant side were higher than those on the dominant side. Conversely, during the kicking phase (P2), the RMS value of the left biceps femoris on the non-dominant side was significantly higher than that on the dominant side. This indicates that the non-dominant side requires higher-intensity muscle recruitment to complete the movement, reflecting inefficient neuromuscular control. This not only impacts striking speed and stability but also increases muscular energy expenditure, thereby affecting overall athletic performance. These findings corroborate the study’s observation: the dominant attacking leg demonstrated higher knee extension power and greater knee extensor group explosive force, resulting in superior striking speed. Previous research indicates that prolonged unilateral training may cause lower-limb muscle imbalances, reducing athletes’ scoring opportunities in competition [52]. This study further confirms that unstable electromyographic patterns during non-dominant side kicking phases may constitute a significant risk factor for technical errors.

In summary, the dominant leg possesses a more efficient neuromuscular control system, enabling higher kicking speeds and more stable movement execution with lower energy expenditure. The non-dominant leg, however, exhibits greater activation demands and control instability, making it a critical focus for enhancing specialized abilities and preventing injuries. It is recommended to incorporate specialized explosive power training, proprioceptive training, and neuromuscular coordination exercises for the non-dominant leg into training regimens to reduce the limitations imposed by unilateral dominance on technical performance.

### 4.4. Kick Momentum: Striking Velocity and Ground Reaction Force Characteristics

Strike velocity is one of the key factors determining scoring in Taekwondo back kick techniques. Under conditions of accurate contact, higher strike velocity generates greater striking force and scoring probability [35]. Research indicates that strike velocity is faster on the dominant side than on the non-dominant side. During the knee lift phase (P1), the knee flexion angle on the dominant side is significantly greater than that on the non-dominant side. Greater knee flexion facilitates rapid knee extension; more complete knee extension leads to faster striking speed [9]. Consequently, the dominant side exhibits higher striking speed than the non-dominant side, particularly during the knee lift phase (P1). The dominant leg demonstrates a larger knee flexion angle, which facilitates a more complete stretch-shortening cycle (SSC). This enables the release of higher speed and force through rapid knee extension during the kicking phase [53]. During the kicking phase (P2), the dominant side exhibits higher peak power output, reflecting greater explosive force. Additionally, the dominant side achieves longer striking distances. This phenomenon indicates superior control and explosiveness during hip abduction and rotation on the dominant side, which enhances kicking range, improves movement execution quality, and increases scoring opportunities. Vertical ground reaction force refers to the equal and opposite force exerted by the ground when an object contacts it. In back kick techniques, the attacking leg primarily generates striking force through knee elevation and hip rotation [9]. Due to the limited range of motion and relatively stable center of gravity height, no significant difference in vertical ground reaction force was observed between the dominant and non-dominant sides [10]. This may be attributed to the minimal changes in center of gravity height during back kick execution and the relatively stable vertical support pattern of the lower limbs. Related studies indicate that in explosive kicking movements, ground reaction forces primarily assist energy transfer through the sagittal plane propulsive component of the supporting leg [40,54].

Therefore, to optimize specialized performance in taekwondo athletes, it is recommended that training focus on enhancing the joint flexion-extension mechanism of the non-dominant side kick, improving proximal force output, and training neuromuscular control efficiency. This approach aims to reduce the limitations imposed by unilateral bias on striking speed and accuracy, thereby enhancing symmetry and technical stability during actual combat.

### 4.5. Limitations

Although this study systematically analyzed bilateral lower limb differences in taekwondo back kicks using 3D motion capture, force plate, and surface electromyography, certain limitations remain. First, the small sample size resulted in slightly reduced statistical power, potentially affecting the generalizability of findings. Second, lateralization determination primarily relied on athletes’ subjective reports; future studies could enhance objectivity by incorporating multi-task functional assessments. Additionally, the electromyography (EMG) recording did not encompass rotational muscle groups, limiting in-depth exploration of body stability and rotational force generation mechanisms. Although paired analyses were conducted for bilateral parameters, asymmetry indices were not calculated. These indices are crucial for quantifying inter-individual functional lateralization levels and should be considered in future studies to enhance analytical rigor. Finally, while violin plots were used to visually represent distribution and significant differences, subsequent studies could incorporate asymmetry index calculations and multidimensional visualization methods to enhance the completeness and practical value of result presentation.

### 4.6. Future Directions and Practical Applications

Future research should incorporate real-time combat conditions to assess biomechanical asymmetries under high-stress scenarios, improving ecological validity. Additionally, integrating imaging modalities such as musculoskeletal ultrasound or musculoskeletal modeling could deepen understanding of joint stress mechanisms.

Practically, findings highlight the need for bilateral lower-limb coordination training to enhance neuromuscular symmetry. Coaches and practitioners should account for bilateral differences and implement targeted training strategies to address these imbalances. Customized strength and neuromuscular training programs can be designed to enhance explosive power output and joint control in the non-dominant leg, particularly during hip and knee joint control in the leg lift and kicking phases. Furthermore, electromyography studies indicate that monitoring and enhancing hamstring activation in the non-dominant limb should be prioritized to improve movement control efficiency. These strategies help optimize kicking performance, enhance bilateral coordination, and reduce overuse injuries caused by load asymmetry, thereby comprehensively elevating competitive readiness and technical stability.

## 5. Conclusions

This study systematically analyzed the kinematic, kinetic, and electromyographic characteristics of both lower limbs during the knee lift and kicking phases of the back kick movement in elite taekwondo athletes, revealing significant lateralization differences. Results indicate superior performance in the dominant leg for hip flexion torque, knee extension power, and striking velocity, reflecting enhanced neuromuscular control efficiency and motor output capacity. Conversely, the non-dominant leg exhibited significantly higher integrated electromyography (iEMG) and root mean square (RMS) values in the biceps femoris and quadriceps femoris muscles, suggesting greater recruitment of motor units to execute the same technical movement. This reflects inefficient neuromuscular recruitment strategies and compensatory patterns. Regarding the support leg, the dominant side’s ankle joint exhibited greater plantar flexion power during the knee-lifting phase, indicating its critical role in kinetic energy generation and ground reaction force transmission. Overall, this study highlights the potential impact of bilateral functional asymmetry in taekwondo back kick techniques on athletic performance and injury risk. It suggests that training should emphasize developing specialized capabilities in the non-dominant leg and optimizing neuromuscular coordination between both legs to enhance movement stability, striking efficiency, and competitive performance.

## Figures and Tables

**Table 1:** Characteristics of Differences in Striking Speed and Vertical Ground Reaction Force.

Indicator	DS	NS	*t*	*p*
Striking speed/(m·s^−1^)	0.67 ± 0.12	0.53 ± 0.08	3.362	0.006
Vertical ground reaction force/BW	100.78 ± 42.81	104.79 ± 42.43	−0.325	0.751

## Data Availability

Data included in article.

## Abbreviations

The following abbreviations are used in this manuscript

DS	Dominant side
NS	Non-dominant side
